# Postmenopausal Osteoporosis: A Literature Review

**DOI:** 10.7759/cureus.29367

**Published:** 2022-09-20

**Authors:** Aayushi Bhatnagar, Aditya L Kekatpure

**Affiliations:** 1 Department of Medicine, Jawaharlal Nehru Medical College, Datta Meghe Institute of Medical Sciences, Wardha, IND; 2 Department of Orthopedic Surgery, Jawaharlal Nehru Medical College, Datta Meghe Institute of Medical Sciences, Wardha, IND

**Keywords:** fractures, estrogen, bone remodelling process, dual energy x-ray absorptiometry, bone formation, bone resorption, postmenopausal osteoporosis, bone mineral density

## Abstract

A substantial proportion of the population of females in India falls in the perimenopausal and postmenopausal age groups. One of the complications associated with older age in women is the weakening of bones and the fall in bone mineral density (BMD). This has a severe debilitating consequence in a woman’s life and leads to reduced quality of life along with a greater incidence of fractures. If the fracture involves the hip or the vertebrae, it can cause immobility and be devastating. Postmenopausal osteoporosis is linked with the deficiency of estrogen that occurs with the cessation of the function of the ovaries as age progresses. The function of estrogen in the bone remodeling process is very well understood after years of research; estrogen plays a part in both the formation of bone as well as the prevention of the resorption of bone. A diagnosis can be made by dual-energy X-ray absorptiometry (DEXA). It is the gold standard and can spot low bone density at particular sites. The treatment options are selected according to the severity and rate of progression and factors pertaining to each patient. All postmenopausal women should be made aware of this disorder, and they should be encouraged to cultivate a healthy lifestyle through the implementation of a proper diet and inculcation of a regular exercise routine. Smoking and drinking alcohol should be limited, and calcium and vitamin D supplementation should be started in all women of the postmenopausal age group with or without osteoporosis. In patients who have been diagnosed with the disorder, pharmacological intervention is done. Drugs should be selected based on their side effects and contradictions. Follow-up is essential, and patient compliance should be carefully monitored. This article attempts to review the existing literature on this very prevalent disorder to spread awareness about it so that all postmenopausal women can take the necessary steps to prevent the weakening of their bones, and deal with its progression.

## Introduction and background

The World Health Organisation (WHO) defines menopause as the complete cessation of menstruation in a woman for one year or more [1]. Perimenopause is irregular menstruation before menopause. Its duration is variable. The average age of Indian women is 46.2 years at menopause [2].

According to the sample registration system statistical report of 2018, 27.7% of the total female population of India falls in the age group of 40 years and above [3]. Given the large population size of older women and the fact that the risk of bone loss and weakening is substantially associated with age as well as menopausal age and its duration, there is a need to understand this disorder and learn about its diagnosis and management. This study aims to review the current literature on this widespread disorder that plagues up to 62% of postmenopausal women [4].

To understand this disorder, we have first attempted to comprehend the components of the human bone. The human skeleton provides a framework for the body. It is composed of a collection of bones secured together by tendons, ligaments, muscles, and cartilage. The bones, which house and protect tissues and organs of the body, are embedded with calcium phosphate crystals and collagen fibers [5]. Bones are composed of a compact component called the cortical bone and a spongy or cancellous compartment, which is known as the trabecular bone and which holds the red marrow. An estimated 80% of the mass of the bone consists of compact bone, and the spongy bone comprises the remaining 20% [6]. The overall strength of a bone is contributed by both the compact and spongy bone [5].

The cortical bone is a network of osteons: dense lamellar units arranged in parallel, concentric circles. A system of Volkmann's canals and Haversian canals connect these osteons and are responsible for nurturing them. The boundary between the osteon and the surrounding extra-osteonal bony matrix is marked by a microscopic structure known as the "cement line" [6-9]. It imparts mechanical strength to the bone [10]. The cortical bone is sandwiched between an outer connective tissue envelope known as the "periosteum" and an inner membrane known as the "endosteum" [6]. The cortical bone is protective and performs mechanical functions [11].

The trabecular bone is a porous, spongy structure that is highly heterogeneous and has varying degrees of anisotropy. It has a higher content of water compared to the cortical bone and a lower percentage of calcium. It contains no blood vessels and is supplied by the surrounding bone marrow. The trabeculated bone tissue stores calcium phosphate crystals, and its primary functions include the transfer of mechanical load from the surface of articulation to the cortex and shock absorption on account of its hydraulic properties [5,6,8,12]. The trabecular bone provides strength and is associated with most metabolic functions [11].

## Review

Osteoporosis

**Figure 1:**
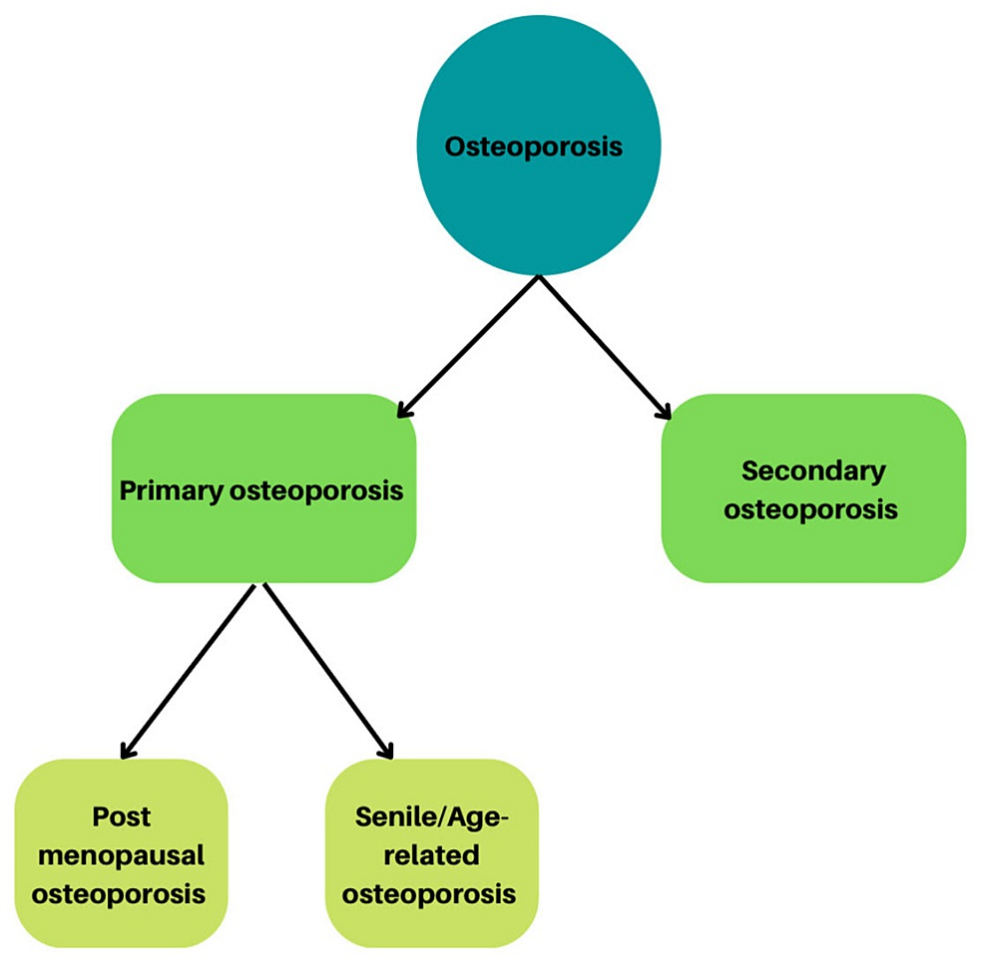
Classification of osteoporosis Osteoporosis is of two types, primary and secondary. Postmenopausal osteoporosis is a type of primary osteoporosis This image is created by the authors

Osteoporosis is a chronic disorder of the skeleton that incapacitates the bones, reflected by a decreased bone mineral density (BMD), predisposing the individual suffering from it to an increased incidence of fractures. It is a debilitating condition with actual physical, social, psychological, and financial consequences [13,14]. Osteoporosis is divided into primary osteoporosis, which results from the normal aging process in humans, and secondary osteoporosis, which occurs due to specific systemic disorders and clinical pathologies [15]. This classification is illustrated in Figure 1.

While osteoporosis can affect anyone of any age group, women of the postmenopausal age group are especially an "at-risk" population to suffer from osteoporosis. In fact, postmenopausal osteoporosis is the most common bone disorder in the developed world [16]. This can be understood better by comprehending the part played by estrogen in the process of maintaining BMD in women.

Bone remodeling

The process of remodeling the bone is dynamic and lasts for life, maintaining the bone mass and quality, limiting the hyper-mineralized bone from accumulating in the body, and keeping check of mineral homeostasis by releasing reserves of Ca2+ and phosphorus. It is necessary to understand that our skeleton does not remain unchanged throughout our life. In fact, our skeleton - up to almost 10% - is reinstated every year [17]. The process of bone turnover involves the tight coupling of bone resorption by osteoclasts and bone formation by osteoblasts [18].

The bone remodeling cycle includes five key steps:

Activation

It marks the commencement of the cycle of bone remodeling, which occurs by recruiting and activating osteoclastic precursor cells from the circulating blood. Activation can either be: a) targeted, whereby osteoclast cells are recruited to a particular location of old or damaged bone, signals of which originate from osteocytes through their extensive signaling dendritic network, and b) nontargeted, which is the activation of osteoclasts not directed to a specific site. This takes place in response to systemic variations in the levels of circulating hormones such as PTH, allowing bony reserves of calcium to be used by the body [17,19-24].

Resorption

Bone mineral dissolution phase: osteoclasts pump protons (H+), which creates an acidic environment that degrades the bony surface. Afterward, matrix metalloproteinases and cathepsin K-like proteases destroy the collagen of the bone matrix. In a healthy bone, this resorption phase is tightly regulated by controlled cell death of osteoclasts to ensure that resorption in undue excess does not occur [17,19,25-28].

Reversal

The coupling of osteoclastic resorption and osteoblastic formation occurs here. Osteoblastic cells are signaled to reach the bony outer plane, and resorption switches to the formation of bone through the function of cytokines such as IL-6 [29-31].

Formation

The process of formation of novel bone corresponds to two functions of osteoblast cells: a) synthesis and secretion of the osteoid matrix, which is abundant in collagen type 1, and b) regulation of the process of osteoid mineralization. The formation is the phase wherein collagen is deposited and mineralizes to form new bone [17,19,23].

Termination

This step marks the apoptosis of osteoblast cells on the completion of bone mineralization, which is regulated by osteocytes by secretion of antagonists of osteogenesis or, in other words, Wnt signaling pathway antagonists, like sclerostin. The osteoblasts that undergo apoptosis can convert into cells lining the bone surface or permanently differentiate into osteocyte cells [32].

Role of estrogen

Menopause-related estrogen deficiency is key to postmenopausal osteoporosis. In the 1940s, it was first suggested by Albright et al. that estrogen deficiency after menopause caused osteoporosis due to impaired bone formation [33]. However, studies that followed demonstrated that was not the case and that the pathogenesis of bone loss was related to rapid bone resorption instead of compromised formation of bone [34-36]. Now, it has also come to light that a deficiency of estrogen causes the acceleration of both processes: increased bone turnover; however, resorption surpasses the augmented formation of bone, which ultimately results in a fall in the bone mass of the body [34,35,37]. The mechanism by which estrogen exhibits its bone-sparing effect is manifold:

Estrogen Suppresses the Production of IL-1 and Tumour Necrosis Factor (TNF) [11]

IL-1 and TNF are potent stimulators of the process of resorption of bone, and they are also well-recognized suppressors of bone formation. They have an immediate impact on osteoblast cells by means of which they indirectly activate mature osteoclasts. They are also potent inducers of additional agents that are associated with the maturation and differentiation of osteoclasts; these include IL-6, macrophage colony-stimulating factor (M-CSF), and granulocyte M-CSF. In vitro, they advance osteolysis, whereas, in vivo, they cause bone loss and a rise in the amount of circulating calcium in the blood. These conclusions are based on results from studies such as one conducted by Pacifici et al., which measured a more significant IL-1 activity by peripheral blood monocytes from 22 osteoporotic patients than by peripheral blood monocytes collected from 14 control subjects [38].

Another study found the raised GM-CSF, IL-1, and TNF-alpha in healthy premenopausal women who had undergone oophorectomy as early as one week after the surgery [39]. It has subsequently been demonstrated that estrogen is also responsible for inhibiting the action of these inflammatory mediators in osteoblasts and stromal cells [40]. Some investigations have found that IL-1 and TNF augment the formation of osteoclast cells by directly promoting the activity of osteoclast precursors [41-44]. Estrogen blocks the activity of these cytokines. T-cells present in the blood are the source of estrogen-regulated TNF, and estrogen is responsible for promptly inhibiting the formation of TNF from these T lymphocytes [45].

Estrogen Indirectly Inhibits the Expression of IL-6

This happens as a result of the blockade of the actions of IL-1 and TNF, which upregulate IL-6 and facilitate its actions. IL-6 acts to activate osteoclast precursors and has a primarily stimulatory effect on the process of osteoclastogenesis. Hence, estrogen has a protective effect on the bone because it inhibits the process of osteoclastogenesis [46].

Estrogen Directly Promotes the Apoptosis of Mature Osteoclasts

Studies conducted on mice have shown that Fas/Fas ligand system is a requisite link in controlling the osteoclast lifespan and is a dominant mechanism of mature osteoclast apoptosis [47,48]. A study has demonstrated upregulation in the expression of Fas receptors in human, mouse, murine and avian-derived osteoclasts during the process of osteoclast activation and differentiation [48]. By promoting the expression of the Fas gene, activation of osteoclasts is directly promoted by estrogen. It was also found by a study conducted in 1996 that transforming growth factor-beta 1 (TGF-beta-1) might be involved with indirectly regulating osteoclast lifespan (by promoting its apoptosis) by estrogen [16].

Estrogen Activates Osteoprotegerin

Osteoprotegerin (OPG) is a naturally occurring member of the TNF receptor superfamily, which acts as a soluble factor in bone mass regulation [49]. In normal and healthy bone remodeling, it primarily originates from the osteoblastic line of cells [11]. OPG acts to neutralize a factor responsible for osteoclastic development, thus, in turn, inhibiting their maturation. A study where OPG was administered in vivo in models involving animals mimicking human bone loss disorders showed that OPG caused downregulation of osteoclastogenesis along with an inhibition of the associated resorption of bone. It also decreased the pathological rise of osteoclasts and their activity [50]. Estrogen is stipulated to promote OPG expression in stromal cells and osteoblast cells [51].

Estrogen Inhibits the Activity of RANK and RANKL

Receptor activator of nuclear factor kappa-B ligand (aka TRANCE/ODF) - a molecule belonging to the family of tumor necrosis ligand and the final effector of osteoclastogenesis, and its receptor RANK - are integral for osteoclast development and activation. They are primary modulators of bone turnover and play a crucial role in bone loss [52]. RANKL is primarily derived from cells of the osteoblastic lineage. These include stromal cells like MSCs, bone lining cells, osteoblasts, and osteoprogenitor cells. These cells show surface expression of both M-CSF and RANKL, which attach to their receptors: RANK and c-fms present on osteoclast precursor cells [53]. They stimulate the formation, activation, and survival of osteoclasts [50]. Estrogen inhibits both RANK and RANKL directly and indirectly. IL-1 and TNF also play a role in upregulating the gene expression of RANKL in stromal cells and osteoblasts; hence, since estrogen inhibits their activity, it indirectly inhibits RANKL activity [54]. RANKL gene expression in stromal cells and osteoblast cells is also stimulated by IL-1 and TNF [11]. This finding is supported by a few studies, such as the one by Eghbali-Fatourechi et al., which reported an elevated expression of RANKL on bone marrow cells such as lymphocytes and osteoblasts in women with postmenopausal osteoporosis as compared to premenopausal control subjects [55].

**Figure 2:**
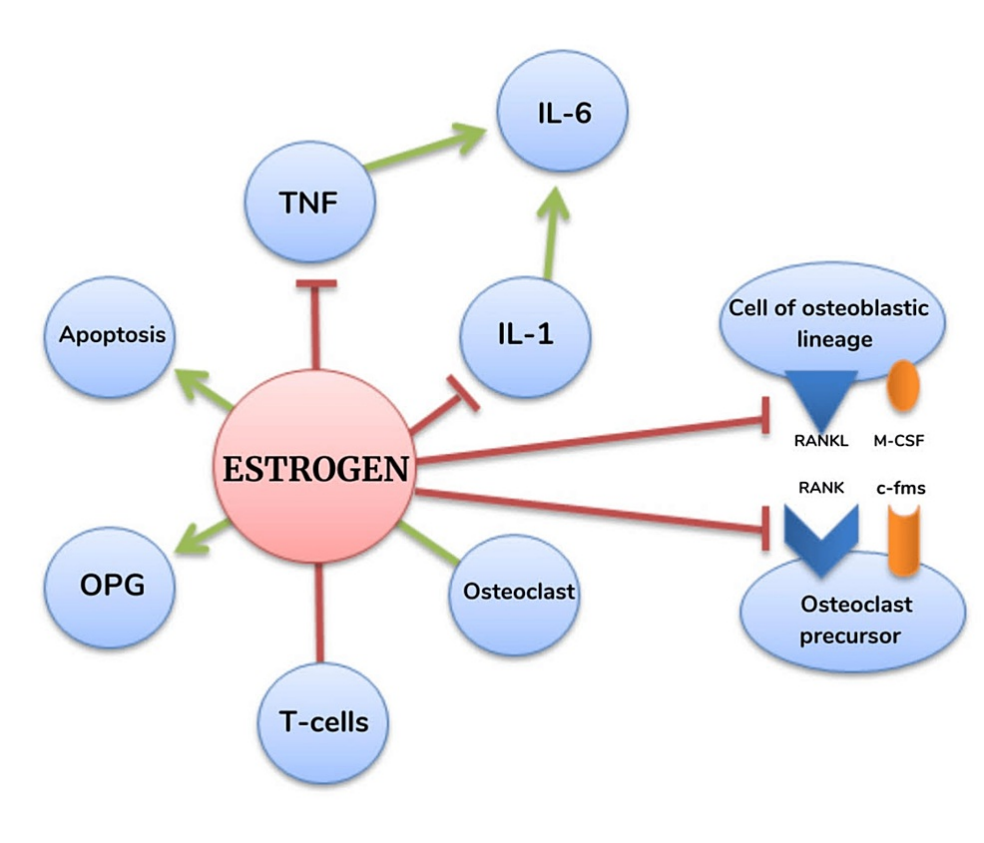
Role of estrogen in the bone remodeling cycle IL-6: interleukin 6; IL-1: interleukin 1; TNF: tumor necrosis factor; OPG: osteoprotegerin; RANKL: receptor activator of nuclear factor kappa-B ligand; RANK: receptor activator of nuclear factor kappa-B; M-CSF: macrophage colony-stimulating factor; c-fms: macrophage colony-stimulating factor receptor The image is created by the authors

Figure 2 depicts the role of estrogen in the process of bone remodeling.

Risk factors

The risk factors corresponding to postmenopausal osteoporosis have been broadly divided into the following groups: racial background, past history, family history, genetic predisposition, medical history, and lifestyle. These have been summarized in Table 1 [56-65].

**Table 1:** Risk factors associated with postmenopausal osteoporosis

Category	Risk factors
Racial identity	White women and Asian women are more prone than black women
Past history	A significant history of hip, vertebrae, tibia, femur, and wrist fractures
Family history	History of fracture of the hip in the family
Genetic predisposition	Polymorphism of WTN16 gene and estrogen receptor genes (ESR1, ESR2)
Medical history	Older age (>65 years), underweight (<125 pounds), eating disorders, delayed menarche, early menopause, type 1 diabetes mellitus, history of bariatric surgery, rheumatoid arthritis, chronic liver disease, kidney failure, inflammatory bowel disease, oophorectomy, COPD/asthma, multiparity, and breastfeeding are hypothesized to affect bone mass. Malabsorption syndromes like celiac disease, low BMD of the neck of the femur, organ transplantation
Drug history	Prolonged use of oral glucocorticoids, thyroid hormone replacement therapy, aromatase inhibitors, anticonvulsants, heparin, warfarin, MPA, anticancer agents, cyclosporine
Lifestyle	Chronic smoking, heavy drinking (two or more drinks on most days), excessive caffeine intake, nutritional deficiency of calcium due to poor intake of milk and its products, immobility or prolonged inactivity, intake of immoderate amounts of saturated fatty acids and decreased consumption of monounsaturated fatty acids (MUFA), low exposure to sunlight causing vitamin D deficiency, fluoride levels ≥2 ppm in drinking water

Diagnosis

Osteoporosis is primarily diagnosed by bone densitometry, which is used to ascertain the density and bone mineral content. It can be done using X-rays, in which case it is called a dual-energy X-ray absorptiometry (DEXA) or DXA, or by means of CT using computer software that scans the hip and spine for determination of their BMD [66]. DEXA is the gold standard. A history of fractures occurring without any significant trauma is usually an indicator of women prone to postmenopausal weakening of bones. Bone densitometry can be used for the early detection of a weakening bone that would benefit from treatment given in due time and can be used to calculate the future risk of fractures [67].

T score

**Figure 3:**
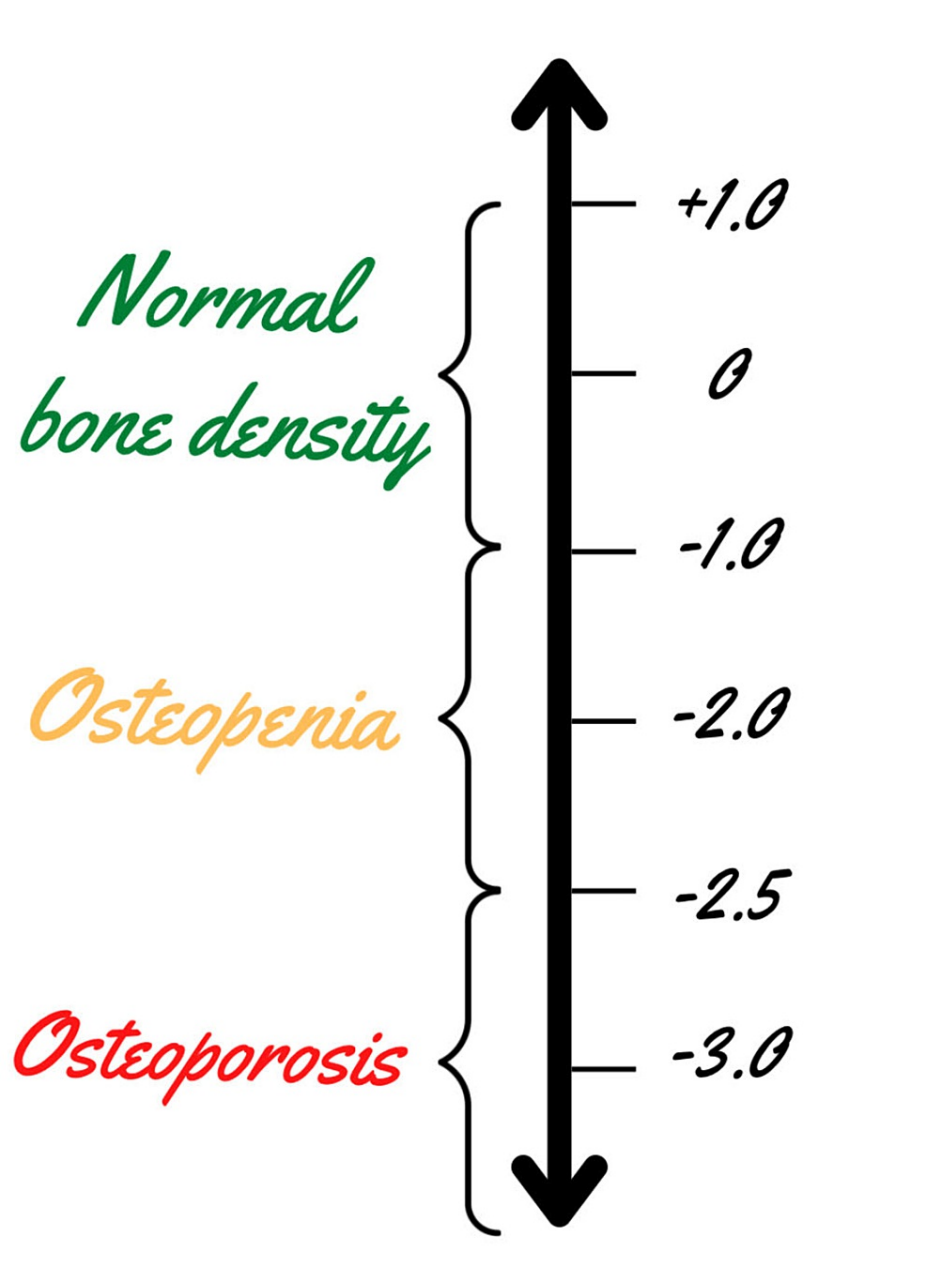
T score grading T score grading is used for the diagnosis of many bone disorders. A grade in the range of 1.0 to -1.0 is healthy, and a grade below -2.5 is diagnostic of osteoporosis This image is created by the authors

The T score measures the standard deviation units from the average value of BMD that is expected in a young adult [68]. Figure 3 depicts the T score grading.

The Z score is another scale that also measures units of standard deviation, which is usually used in children and adolescents. It is calculated in a way similar to the calculation of T scores, but the comparisons are made with someone of the subject's age, sex, height, weight, and race [66].

The World Health Organization defines osteoporosis on the basis of the T score as a standard deviation of 2.5 or more below the young adult's mean [66].

Management

The management of postmenopausal osteoporosis can be broadly classified into general management and pharmacological therapy.

General Management

It includes lifestyle modifications like following a carefully planned diet, exercising regularly, avoiding habits such as smoking and consumption of alcohol, taking calcium and vitamin D supplements, as prescribed by the physician, and avoiding falls to prevent fractures and injuries.

It is recommended for all postmenopausal women to maintain a balanced diet rich in calcium and proteins. The current NOF guidelines recommend a calcium consumption of 1200 milligrams daily for women. Calcium is an essential bone of mineralized tissues in the body, and its adequate intake helps contributes towards maintaining bone health, mediating contractions of muscles and blood vessels, the transmission of nerve impulses, and plays a role in intracellular and extracellular signaling [69]. Adequate protein intake (both plant-based and dairy-based) helps prevent age-associated bone loss and muscle loss. The RDA of protein in postmenopausal women is 1-1.2 g/kg/day [70]. A higher intake of fruits, whole grains, and nuts and a reduced intake of processed meat, candy, and saturated fats is associated with increased BMD at several sites [71].

Immobility and prolonged inactivity are risk factors for postmenopausal osteoporosis. Bone mass can be improved, and fracture risk can be reduced by exercise, including weight-bearing. Walking is associated with the improvement of hip BMD and postural stability [72-74]. A study conducted by Roghani et al. found that balance improved in women wearing a weighted vest while walking than in those who were not wearing one [75]. A combination of weight-bearing exercise with resistance training is beneficial in people who have suffered bone loss, and this helps to improve functional performance as well as spinal and hip bone density [76,77]. Yoga, under a trainer's guidance, can improve posture and increase bone density [78,79].

Since both smoking and alcohol are risk factors for osteoporosis, their use should be addressed in postmenopausal women [13].

Calcium and vitamin D supplements are first-line treatments for postmenopausal osteoporosis. In women without adequate dietary habits and in patients who are on drugs with reduced secretion of gastric acid (as acid is required for the absorption of calcium carbonate), it is advised to administer calcium citrate maleate. Nearly 70% of the population in India suffers from a deficiency of vitamin D [80]. Vitamin D is supplemented through cholecalciferol - 60,000 units given once every one to two months - and this is recommended in all postmenopausal women, with or without osteoporosis. Calcitriol or active vitamin D is usually not prescribed except in cases of renal impairment.

Falls can be prevented by improving the safety of homes. Avoiding lowly lighted surroundings, fixing slippery surfaces, stairs, and carpets, carefully treading on wet surfaces, wearing rubber-soled shoes that are low-heeled and skid-free, standing up slowly, and using assistive devices to walk, if needed, are recommended [81].

Pharmacological Treatment

Usually, pharmacological intervention is considered in high-risk cases such as in postmenopausal women with BMDs corresponding to a T score of ≤2.5 at the regions of the spine, neck of femur, lumbar vertebrae or the region of the hip, in postmenopausal women with hip or a vertebral osteoporotic fracture, or based on Fracture Risk Assessment Tool (FRAX) calculations of 20% risk of a significant fracture at the shoulder, wrist, hip, or spine, or a 3% risk of a hip fracture in postmenopausal women with T scores ranging from -1.0 to -2.5 with a 10-year risk.

What is a FRAX Calculator [82-84]?

FRAX stands for Fracture Risk Assessment Tool. It is the most widely used tool by doctors to assess the risk for significant fractures in the future due to osteoporosis.

It is a questionnaire that measures said risk with questions focusing on the age, weight, and gender of the individual, history of smoking (if any), amount of alcohol intake, history of fractures, use of glucocorticoids (which can cause loss of bone), comorbidities such as rheumatoid arthritis, and the calculated BMD. A score is then calculated by a doctor based on the answers to the above questions, which determines a 10-year risk of any significant fracture that could be caused due to osteoporosis and a 10-year risk percentage of a hip fracture.

A score of >5 for a hip fracture at years 70 and above indicates the need for pharmacological intervention and lifestyle modifications, and a score that is lesser but at a younger age warrants a physician's supervision and could require treatment [82].

DEXA remains the gold standard because FRAX has a few drawbacks, e.g., it can only be used in untreated patients not currently on any treatment for osteoporosis [85]; moreover, it does not take into account the fact that the risk for a fracture tends to increase post a prior fracture nor does it take into consideration the change in the dose-response relation between a single and multiple fractures [86,87]. It fails to acknowledge the dosage and duration of the uses of glucocorticoids [88], and it only scores the BMD of the femoral neck and does not take into account other sites, such as the lumbar vertebrae [85].

Pharmacological therapy can be broadly classified into hormone replacement therapy with estrogen, the use of anti-resorptive agents, and the administration of anabolic agents.

Estrogen is a distinctive compound that has both anti-resorptive as well as anabolic effects on the bone [89]. Anti-resorptive agents include denosumab and bisphosphonates. Anti-resorptive agents reduce the risk of fractures by decreasing bone loss, whereas anabolic agents help achieve the same by increasing the formation of new bone.

Estrogen Therapy

Since the pathophysiology of postmenopausal osteoporosis is well understood, it seems appropriate to consider the use of estrogen as a therapeutic option in both perimenopausal and postmenopausal women. In a study conducted by the Women’s Health Initiative in 2002, it was brought to light that using estrogen had many harmful effects, which outweighed its benefits. It found that using estrogen hormone replacement for five years or more multiplied the risks of congestive heart disease (CHD), venous thromboembolism, stroke, and carcinoma breast, and it could even cause cholecystitis [90]. These findings were revised later to obtain a more refined comprehension of the risks and benefits of menopausal hormone therapy [91,92]. The benefits of estrogen therapy are significant in women who have an estrogen deficiency due to early menopause or a primary ovarian insufficiency. Menopausal hormone therapy (MHT) can help solve additional painful vasomotor symptoms in women experiencing surgical menopause. It can also resolve genitourinary symptoms such as vaginal dryness, vulval itching, increased frequency of urination, dyspareunia, nocturia, urge incontinence, and UTIs [93]. It has been found that HRT can maintain and even increase BMD at all locations in postmenopausal women, like the femoral neck, lumbar vertebra, and forearm [94]. Postmenopausal females below the age of 60 years, who are prone to fractures, or are within 10 years of menopause, can be started on estrogen therapy as the first line of treatment [95]; however, it is not preferred in women above 60 because of the risk of acquiring breast cancer [93]. In women with intact uteri, progesterone is recommended along with estrogen, as long-term use of unopposed estrogen can cause endometrial hyperplasia and cancer. In women without uteri, estrogen can be given alone [93].

Anti-Resorptive Agents

These include bisphosphonates, denosumab, selective estrogen receptor modulators (SERM), and calcitonin. Bisphosphonates are highly efficacious drugs that are often considered a primary treatment modality for postmenopausal women at an increased risk of fractures [12]. Some bisphosphonates available for treatment in India are listed in Table 2 [96-101].

**Table 2:** Bisphosphonates available in India for the treatment of osteoporosis

Drug	Action	Side effects	Contraindications	Dosage
Zoledronic acid	It reduces the risk of vertebral fractures by 70%, hip fractures by 41%, vertebral fractures by 77%, clinical fractures by 33%, and non-vertebral fractures by 25%. Improves BMD and bone metabolism markers	Renal impairment, osteonecrosis, dyspnoea, pain in the muscles, bone, or joints, headache, anemia	Not to be used in pregnancy or during breastfeeding, contraindicated in patients with pre-existing kidney disease, cancer, blood disorders, dental problems	4 mg IV once a year
Alendronate	Decreases the occurrence of fractures of the spine and femur by an estimated 50% over a period of three years in patients who have had prior vertebral fractures. Decreases the occurrence of vertebral fractures by approximately 44% in patients with no history of vertebral fractures	Muscle spasms, swelling of joints and limbs, eye pain, chest pain, blisters on the skin or peeling of skin, painful gums	Vitamin D deficiency, atrial fibrillation, hypocalcemia, cardiac achalasia, oesophageal ulcer, inflammation surrounding the tooth	70 mg weekly
Ibandronate	Decreases the occurrence of fractures of the vertebrae by 50% over three years and data suggests its efficiency in reducing non-vertebral fractures	Gastric, oesophageal ulcers, heartburn, allergic reactions, painful bone, joint, or muscle	Several renal impairments, gastritis, achalasia cardia, hypocalcemia, bedridden patient, Barrett's esophagus, in case of an invasive dental procedure	150 mg monthly
Risedronate	Decreases the rate of occurrence of vertebral fractures by 41% to 49% and non-vertebral fractures by 33% to 39% in patients with a prior history of fractures	Gastric, oesophageal ulcers, heartburn, allergic reactions, painful bone, joint, or muscle	Hypocalcemia, achalasia cardia, gastritis, oesophageal ulcer, inflammation in tissues surrounding the tooth, patients undergoing an invasive dental procedure	35 mg weekly

Denosumab is a human monoclonal antibody that binds with and inhibits cytokine RANKL. It inhibits the maturation of osteoclasts and their survival, thereby lowering the resorption of bone [102]. It can be administered in patients with CKD. It is usually given once every six months via subcutaneous injection. Some common side effects of denosumab include hypersensitivity reactions, hypocalcemia, and excess suppression of bone turnover. It has been found that denosumab usage causes a reduction in hip fractures by up to 40% and leads to a 68% decrease in vertebral fractures [103].

Selective estrogen receptor modulators (SERMs) are synthetic drugs that are nonsteroidal and mimic the effects of estrogen on the skeleton and the CVS without any harmful effects on the endometrium and breast. The prototype drug is raloxifene, which is known to decrease risks associated with fractures of the vertebrae and breast cancer [104,105]. However, raloxifene has side effects such as an increased risk of venous thromboembolism and the occurrence of hot flashes [104]. FDA has approved the combination of bazedoxifene (another SERM) and conjugated estrogens to treat osteoporosis without causing hot flashes [106].

Calcitonin is a polypeptide that takes part in the metabolism of calcium and phosphorus, reducing overall bone turnover [107]. Calcitonin is not advised as a therapeutic agent in osteoporosis because of the associated risk of cancer with prolonged use [108].

Anabolic Agents

These include teriparatide. Teriparatide is a synthetic form of parathyroid hormone; its bioactive part is composed of 34 amino acids. In 1934, Albright et al. [109] made an observation that PTH was responsible for both the formation of bone and its destruction by osteoclasts. Subsequently, it was discovered that in small doses given continuously over a long period, parathyroid hormone exerted a net anabolic effect on the bone and hence could be helpful in osteoporosis [110].

Findings from these studies and more clinical trials subsequently found that at an FDA-approved dose of 20 μg/day, teriparatide reduces the incidence of vertebral fractures by 65% and the risk of non-vertebral ones by 53% [111]. Teriparatide is linked with the risk of osteosarcoma in animal trials; however, no such evidence of increased incidence of the same has been found when tested in humans. It is contradicted in pregnancy, patients of the pediatric age group, patients with Paget's disease other metabolic bone disorders apart from osteoporosis, patients with already existing hypercalcemia, bone cancer, and a history of bone radiation therapy [112]. A PTH-related peptide analog named abaloparatide has been approved by the FDA, which might offer some advantages over teriparatide [108].

**Figure 4:**
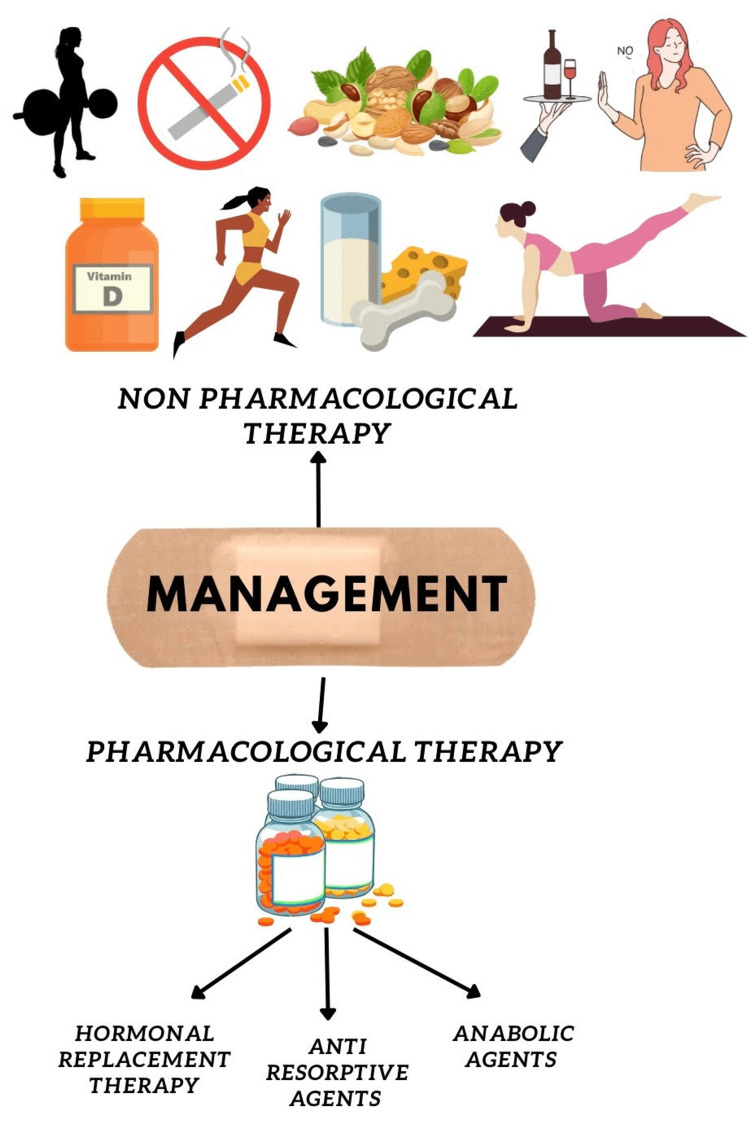
Management modalities of postmenopausal osteoporosis Postmenopausal osteoporosis can be managed by bringing about small but significant changes in one’s lifestyle. Exercise, yoga, consumption of milk and its products, and stopping smoking and alcohol consumption can be very beneficial. If the disease has already progressed, the pharmacological intervention includes hormonal replacement, anti-resorptive agents, and anabolic agents This image is created by the authors

Figure 4 depicts a summary of the management of postmenopausal osteoporosis.

## Conclusions

Postmenopausal osteoporosis is a silent disease that is very prevalent worldwide, as well as in India. It is a chronic condition that is asymptomatic, and its progression is slow. After years of research, we have finally discerned its pathogenesis and etiology. It is also evident what its risk factors are, and its diagnostic tools are now widely used. Though it can be debilitating for those who suffer from it, postmenopausal osteoporosis can be managed by both pharmacological and non-pharmacological intervention. Without proper diagnosis and treatment, it can cause a severe fall in the quality of life. Efforts are needed to be made to ensure that all postmenopausal women in India are made aware of the lifestyle modifications that need to be made during the perimenopause and postmenopause periods to help prevent this disorder, and treatment options should be made available to all patients at a minimal cost.
